# Factors influencing long-term persistence of anti-HBs after hepatitis B vaccination

**DOI:** 10.1038/s41541-022-00596-5

**Published:** 2022-12-26

**Authors:** Marco Fonzo, Chiara Bertoncello, Andrea Trevisan

**Affiliations:** Department of Cardiac Thoracic Vascular Sciences and Public Health, University of Padova, Padova, Italy

**Keywords:** Protein vaccines, Hepatitis B, Epidemiology

## Abstract

Long-term immunity after HBV vaccination is still debated. When assessing immune persistence, several variables must be considered, the clear definition of which is crucial. Our aim was to assess protection 10–20 years after primary vaccination and to estimate the effect of age at first dose, sex and time elapsed between doses on long-term protection. We conducted a retrospective cohort study between January 2004 and December 2020. Antibody titres above 10 IU/L were considered protective. Geometric mean titres (GMT) were calculated. The effect of the above variables on long-term protection was assessed by logistic regression analysis. Included participants were 9459. Among those vaccinated during infancy, GMT gradually increased from 11 IU/L (first dose in 1st trimester of life) to 68 IU/L (4th trimester), while the proportion of individuals <10 IU/L remained stable between 1st and 2nd trimester (51%) and it decreased substantially in 3rd (28%) and even more so in the 4th (18%). A one-month delay in first and third dose administration was correlated with a −16% (AOR: 0.84; 95% CI: 0.78–0.91) and a −11% (AOR: 0.89; 95% CI: 0.85–0.94) risk of a titre <10 IU/L, respectively, ~20 years after immunisation. In contrast, similar changes do not comparably affect vaccination in adolescence. The start of vaccination at the third month of age is a compromise between the development of acceptable immunogenicity and the need to protect the infant as early as possible. However, the chance of slightly delaying the vaccine administration within the first year of life may be considered given the impact on long-term persistence of anti-HBs.

## Introduction

Viral hepatitis is one of the most important infectious diseases and it is indicated to be responsible for about 78% of cases of primary liver cancer. In 2019, the World Health Organization (WHO) estimated that hepatitis B (HBV) infections resulted in 820,000 deaths globally, mostly from cirrhosis and hepatocellular carcinoma, with 296 million people living with chronic disease and 1.5 million new infections each year. The burden of HBV is highest in the Western Pacific Region and Africa, where 116 million and 81 million people are chronically infected, respectively. In Europe 14 million people are infected, although in the last decades a downward trend in the rate of acute cases was observed, mainly due to the extensive implementation of immunisation practices^1^. In Italy, the incidence rate of acute HBV cases decreased from 5 per 100,000 inhabitants in 1990 to 2 per 100,000 ten years later. In 2021, only 89 new cases were reported, with an incidence of 0.18 per 100,000 inhabitants^2,3^.

Since the 1980s, safe and highly effective vaccines against HBV are available. In 1992, the WHO recommended the inclusion of the HBV vaccination in all nationwide immunization programs, however, the long-term persistence of immunity following vaccination and the eventual need for a booster dose are still debated, as the collection of evidence in this regard is still ongoing^4^.

When assessing the immune persistence of the HBV vaccine, a number of variables must be taken into account, the clear definition of which is crucial. For instance, quite often the expression “infant vaccination” indicates the first dose administration within the first year of life at any age, but a difference of few months may result in a different vaccine-induced antibody production^5^.

The aim of this study was to assess the protection against HBV after ~10–20 years from the completion of the primary vaccination course and to estimate the potential impact of age at the first dose, sex and length of time between doses on long-term protection in a low endemicity country.

**Table 1:** Characteristics of the study population.

		Cohort 1		Cohort 2	
		*n* = 5485		*n* = 3974	
		*n*	%	*n*	%
Sex	Male	1953	36%	1236	31%
	Female	3532	64%	2738	69%
		mean	SD	mean	SD
Age at 1st dose (months)		3.04	0.80	136.41	4.32
Time 1st–2nd dose (days)		54.61	9.79	39.21	9.44
Time 2nd–3rd dose (months)		7.18	1.08	5.72	0.97
Follow-up duration (months/years)		19.35	0.97	9.54	1.82

*SD* standard deviation.

## Results

### Descriptive findings

**Fig. 1:**
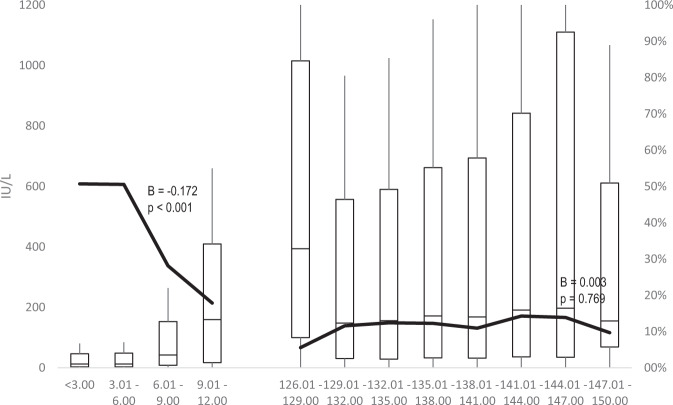
Long-term anti-HBs titre (boxplot, left *y* axis) and proportion of individuals below 10 IU/l (black line, right *y* axis) by age (months) at the first dose vaccine administration.

A total of 9459 participants were included in the study. Cohort 1 included 5485 participants, of which 64% females, while Cohort 2 included 3974 participants, of which 69% females. They all were vaccinated with a three-doses schedule between May 1990 and August 2003. The duration of the follow-up was of 19.35 months in Cohort 1 and 9.54 years in Cohort 2. None of the participants tested positive for HBcAb or HBsAg. Details on the two studied cohorts are reported in Table 1. As of participants who received the vaccine during infancy (Cohort 1), the GMT increased from 11 IU/L (first dose administration in the 1st trimester of life) to 68 IU/L (4th trimester). Conversely, the proportion of individuals with a titre below 10 IU/L remained stable between 1st and 2nd trimester of life (51%), while substantially dropped in 3rd trimester (28%) and even further in the 4th (18%), as reported in Table 2 and Fig. 1. As of Cohort 2, the GMT ranged between 94 and 178 IU/L; the proportion of participants with a titre below 10 IU/L ranged between 6% and 14%.

**Table 2:** Long-term anti-HBs titre and proportion of individuals below 10 IU/l by age at the first dose vaccine administration.

Age at 1st dose	*n*	Antibody titre		<10 IU/L		
		GMT	GSD	%	95% CI	
Cohort 1 (total)	5485	10.38	7.53	50.3%	49.0%	51.7%
1st trimester of life	3217	10.57	5.51	50.7%	49.0%	52.4%
2nd	2208	11.01	5.78	50.5%	48.5%	52.6%
3rd	32	30.64	5.15	28.1%	14.9%	45.1%
4th	28	67.57	6.54	17.9%	7.2%	34.8%
Cohort 2 (total)	3974	101.46	6.65	12.2%	11.2%	13.2%
10 y 3rd trimester	18	178.34	5.72	5.6%	0.6%	23.2%
10 y 4th	613	94.37	6.36	11.6%	9.2%	14.3%
11 y 1st	1113	97.40	6.31	12.4%	10.6%	14.4%
11 y 2nd	800	103.76	6.56	12.3%	10.1%	14.7%
11 y 3rd	797	102.96	6.86	10.9%	8.9%	13.2%
11 y 4th	400	106.18	7.41	14.3%	11.1%	17.9%
12 y 1st	202	118.31	7.66	13.9%	9.6%	19.1%
12 y 2nd	31	104.22	6.98	9.7%	2.8%	23.6%

*GMT* geometric mean titre, *GSD* geometric standard deviation.

### Effect of 1st and 3rd dose delay on antibody persistence

**Fig. 2:**
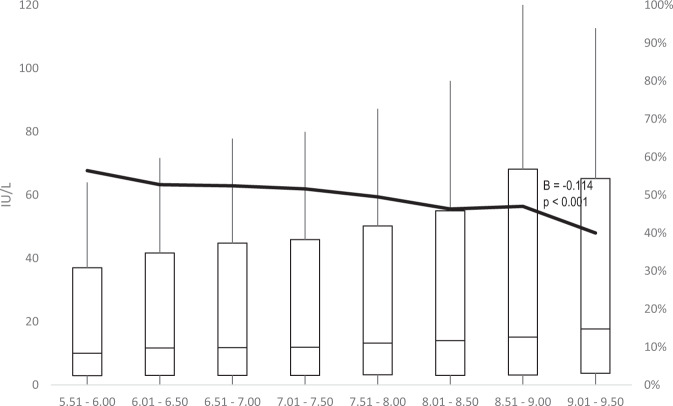
Long-term anti-HBs titre (boxplot, left *y* axis) and proportion of individuals below 10 IU/l (black line, right *y* axis) by time interval (months) between the administration of the second and the third dose in the Cohort 1.

**Fig. 3:**
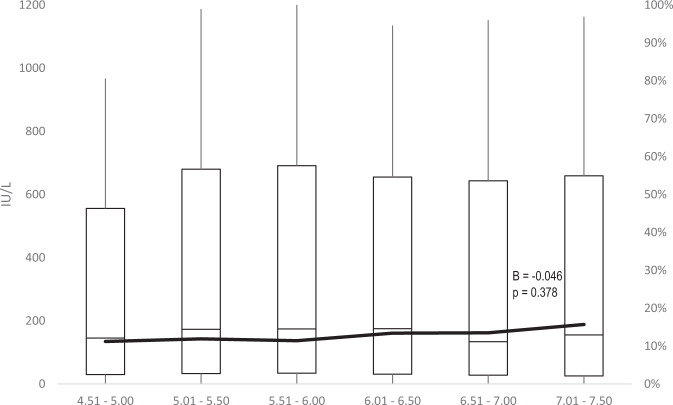
Long-term anti-HBs titre (boxplot, left *y* axis) and proportion of individuals below 10 IU/l (black line, right *y* axis) by time interval (months) between the administration of the second and the third dose in the Cohort 2.

Results of the logistic regression showed that in Cohort 1 a one-month delay in the administration of the first dose and a one-month delay in the third dose administration were correlated with a −16% (AOR: 0.84; 95% CI: 0.78–0.91) and a −11% (AOR: 0.89; 95% CI: 0.85–0.94) risk of having a titre below 10 IU/L, respectively (Table 3). No association was noted with sex. On the contrary, in Cohort 2 the female sex was associated with a reduction in risk of having a titre below the threshold (AOR: 0.66; 95% CI: 0.54–0.81), while no correlation was noted with other variables investigated. Considering the time interval between the administration of the second dose and the third one, the GMT showed a substantial increase in Cohort, 1 in accordance with the extension of the time span at issue (Table 4 and Fig. 2), raising from 8 up to 14 IU/L. The proportion of individuals with a titre <10 IU/L gradually decreased from 56% in individuals who received the third dose 5.5–6 months after the second one to 40% (9–9.5 months between second and third dose). In Cohort 2, the GMT remained substantially stable around 100 IU/L, while the percentage of participants with <10 IU/L slightly increased from 11 to 16% (Fig. 3).

**Table 3:** Logistic regression.

	Cohort 1			Cohort 2		
	AOR	95% CI		AOR	95% CI	
Female sex	1.01	0.90	1.13	0.66	0.54	0.81
Age at 1st dose (1 month increase)	0.84	0.78	0.91	1.00	0.98	1.03
Time 1st–2nd dose (days)	1.01	1.00	1.01	1.01	1.00	1.02
Time 2nd–3rd dose (months)	0.89	0.85	0.94	0.95	0.86	1.06
Follow-up duration (years)	0.88	0.76	1.01	1.06	0.80	1.41

*AOR* adjusted odds-ratio; adjustment for date at the first dose and date at the analysis not shown.

The effect of studied variables on the risk of having an antibody titre below 10 IU/l.

**Table 4:** Long-term anti-HBs titre and proportion of individuals below 10 IU/l by time interval between the administration of the second and the third dose.

Time 2nd–3rd dose (months)	Antibody titre		<10 IU/l		
	GMT	GSD	%	95% CI	
*Cohort 1*					
5.51– 6.00	8.14	5.40	56.4%	51.4%	61.4%
6.01–6.50	9.52	5.31	52.7%	49.8%	55.7%
6.51–7.00	10.10	5.66	52.4%	49.3%	55.4%
7.01–7.50	10.42	5.67	51.6%	48.3%	54.9%
7.51–8.00	11.72	5.39	49.5%	46.1%	52.8%
8.01–8.50	12.73	5.77	46.3%	42.1%	50.5%
8.51–9.00	13.83	6.27	47.0%	41.5%	52.5%
9.01–9.50	13.94	5.83	40.0%	32.2%	48.3%
*Cohort 2*					
4.51–5.00	93.17	6.59	11.2%	8.4%	14.6%
5.01–5.50	104.47	6.52	11.9%	10.3%	13.7%
5.51–6.00	105.73	6.55	11.4%	9.5%	13.4%
6.01–6.50	101.53	6.93	13.4%	10.7%	16.5%
6.51–7.00	95.45	6.84	13.5%	9.5%	18.5%
7.01–7.50	87.98	8.34	15.7%	9.8%	23.5%

*GMT* geometric mean titre, *GSD* geometric standard deviation.

## Discussion

Most of participants vaccinated during adolescence, namely Cohort 2, showed a protective anti-HBs titre (only 12.2% with anti-HBs titre <10 IU/L), while 50.3% of those vaccinated in the first year of life showed a titre below the established threshold. This finding is consistent with the current evidence concerning the long-term persistence of seroprotection levels in vaccinated subjects. Several studies concluded that the vaccine-induced immunity in individuals vaccinated during adolescence is higher in terms of both antibody titre and proportion of individuals with a titre above 10 IU/L, regardless of the time elapsed between the last dose and the serological test^2,6–12^.

Nevertheless, this was not the main purpose of our study. As highlighted by Osiowy^4^, we decided to go beyond the expressions “infant or adolescent vaccination” by dissecting the actual age at the vaccine administration and considering the trimesters of life. This was feasible due to the presence of a consistent number of deviations from the recommended vaccination schedule, which attracted our attention.

Our investigation highlighted how a restrained delay in vaccine administration did not show a substantial effect in adolescents, whereas this has a considerable impact during the first year of life. GMT increased approximately three-fold in the third trimester and even six-fold in the fourth trimester. As shown by logistic regression, each month of delay within the first year of life is associated with a 16% reduction in having an anti-HBs titre below 10 IU/L approximately 20 years after primary vaccination. The time it takes for a full maturation of the antibody response to occur has been well demonstrated by the observation of low antibody levels, e.g., after measles vaccination, when the vaccine is given at 9–11 months compared to infants vaccinated at >12 months of age. On the other hand, immunisation responses before 6 months of age have shown low immunogenicity and this phenomenon appears to be only partly attributable to the presence of antibodies of maternal origin^13,14^. However, recent evidence showed that specific memory B cells are more affected by age than serum antibody levels, so that immunoglobulin levels in plasma might not reflect the actual status of B-cell compartment in real time^15,16^.

Overall, the immune system in childhood is characterized by an impaired T-cell function, by lower interactions between B and T cells, by a reduced assortment of immunoglobulins and by a low-affinity antibody response^17^. Response in the infant is predominantly IgM, as a sign of a defect in isotopic variation due to limited T-cell helper activity. Repeated antigenic administrations are usually necessary to allow early B cells to receive sufficient impulses from T cells to switch from IgM to IgG synthesis^18^.

As shown by results of the logistic regressions, not only the age at the first dose, but also an extension of time between 2nd and 3rd dose is correlated with a lower risk of titre <10 IU/L in infants, in particular a 11% reduction per month, while no correlation was noted with the time between the 1st and 2nd dose. Our results describe a well-documented phenomenon. This finding may be explained by a more mature immune system in older infants but also a maturation of the immune memory during the longer interval as shown for polio, hepatitis B and hepatitis A vaccine, both in children and adults^19–21^.

As early as 1989, a study conducted in Venezuela on a population of Yucpa Indians of all ages showed a direct relationship between response to the HBV vaccine and the timing of the third dose. In particular, those receiving the third dose 7 or more months after the first one developed antibody titres two-fold higher compared with those receiving a standard three-dose schedule at 0, 1, and 6 months. Response was reduced by 30% in those who received the third dose 1 month earlier or more^22^. A more recent meta-analysis conducted by Schönberger and colleagues on determinants of long-term protection after HBV vaccination showed that a gap time lower than 6 months is associated with a lower long-term persistence of anti-HBs compared with a gap between 6 and 8 months, but did not find any association with time intervals higher than 8 months^23^. Our study instead suggests a gradual increase in the GMT and a gradual decrease in the proportion of individuals with titre <10 IU/L, according with the extension of the gap time between the last and preceding dose.

Speaking of vaccination in general, the ideal differentiation pathway of B cells towards memory B cells could benefit from a ‘early prime – later boost strategy’, as stated by Siegrist and Aspinall. On the one hand it makes sense to define the earliest time for the first dose administration, according with the efficiency of the infant’s immune response. However, on the other hand it is important to administer the last dose after a sufficiently long interval for affinity maturation to occur^5^.

On the basis of the results of multivariable analyses in the two cohorts, females showed a lower risk of having a titre below 10 IU/L, but only among adolescents. Literature generally agrees on this point^24–27^, although in some cases a gender-related difference was observed independently from the patients vaccination cohort^10,28^. This may arise by the different effect that sex hormones have on the immune system. In fact, oestrogens play an important role in adaptive immunity by enhancing humoral responses, B cell differentiation and immunoglobulin production^29^. Oppositely, testosterone inhibits the production of IgG and IgM from B lymphocytes and reduces IL-6 synthesis from monocytes^30^.

In Italy, HBV epidemiological scenario changed considerably in recent years, with a decrease in both incidence and prevalence of markers of infection. The reasons for these changes lie in both improved socio-economic conditions and specific interventions, such as the promotion of active immunisation and prophylaxis of exposed individuals, the increasing use over the years of disposable medical equipment and greater care in invasive diagnostic procedures and surgery and screening tests in blood donors. In addition, screening of HBsAg in pregnant women has been introduced to identify new-borns who need both active and passive immunity induction by concomitant administration of the vaccine and 200 units of immunoglobulin within 24 hours from delivery^31^. The presence of a National Surveillance System (SEIEVA) since 1985 has been of paramount importance for the early identification of cases of infection and implementation of appropriate containment measures^32^. As a result of all implemented measures, in Italy during 2021, 89 cases of hepatitis B were reported (incidence 0.18/100,000), with zero cases reported in the 0–14 age group. Over the past 10 years, the incidence in this age group has fluctuated between 0 and 0.1/100,000. The most affected age group consistently remains the 35–54 age group. It should also be noted that the most frequently reported exposure are beauty treatments such as manicures and pedicures but also piercings and tattoos (28% of cases), followed by promiscuous sexual behaviour (23%). The risk of nosocomial transmission (hospitalisation, surgery, haemodialysis or blood transfusion) is reported by 13.8% of cases, while the proportion due to exposure with cohabitants is 9%^3^.

It is worth mentioning that a delay in the administration of the third dose has been *de facto* already put into place in Italy. Indeed, despite the National Vaccine Prevention Plan 2017–2019 recommends HBV vaccination according to the 3rd–5th–11th month schedule, usually in combination in the hexavalent formulation^33^, in some Italian Regions such as Tuscany or Veneto the third dose is given at 13 months of life^34^. The reason behind this choice is essentially logistical, in order to administrate the hexavalent vaccine in the same occasion with the meningococcal C one^35^. This association is well tolerated, safe and effective, and current evidence suggests the absence of a mutual interference between the two vaccines that might lead to an altered immune response^36,37^.

This study has some limitations. For instance, no data were retrieved about the type of vaccine received at the time of primary immunisation. Vaccination against HBV was introduced in Italy in 1983, initially for people belonging to high-risk groups. However, the switch from the plasma-derived vaccine towards the recombinant one, although in a gradual manner, occurred in the mid-1980s, so that it is reasonable to supposed that all participants involved in our study were vaccinated with a full course of recombinant type^38^. Until combined vaccine formulations became available in 2000, two main recombinant vaccines against HBV were employed for both cohorts of children and adolescents: Engerix-B^®^ (10 μg dose), the most used from the 1991 to 1993, and Recombivax HB^®^ (5 μg dose) for paediatric use^39^. We were not able to assess the reason of vaccination delays compared with the recommended schedule whether they were the results of a medical advice or only parental vaccine hesitancy, in particular as regards participants in Cohort 1. Data regarding other factors and behaviours that may adversely affect HBV vaccine immunogenicity, including obesity, smoking habits, or concomitant diseases^27,40^ were not collected.

Many factors influence the choice of age at which vaccines are administered, e.g. the potential interference with immune responses by antibodies passively transmitted by the mother; the age at which the risk for the disease or vaccine-related complications is highest; the ability to respond to the vaccine at different ages. The start of vaccination at the third month of age is the result of a compromise between the need to defend the infant as early as possible and the knowledge that the later the start of vaccination, the greater the immunogenicity and therefore the greater the effectiveness. However, the chance of slightly delaying the HBV vaccine administration within the first year of life, compared with the current schedule, may be taken into account given the long term persistence of anti-HBs, as demonstrated in this study. In contrast, similar changes do not affect vaccination in adolescence in a comparable way. Although considerations arisen from this study are not intended to be an immediately ‘ready to use’ information for public health decision makers, they may lay the ground for future speculation, considering the perspectives from both an immunological and epidemiological point of view in low endemicity countries. Last but not least, studies assessing the cost-effectiveness of potential hypotheses in this regard would be desirable.

## Methods

### Study design

We conducted a retrospective cohort study, enrolling students and resident doctors attending the Medical School of Padua University, Italy. Data on age, sex, date of vaccine administration, as well as blood samples, were collected at the time of the first occupational health surveillance medical examination between January 2004 and December 2020. Routine detection of anti-HBs titre in serum before employment has been recommended for healthcare workers and students attending healthcare-related courses by the National Vaccine Prevention Plan (PNPV). The following inclusion criteria were considered: being born in Italy from HBsAg-negative mother, having received a three dose schedule vaccination against HBV as witnessed by a certificate issued by the Public Health Office, age at time of enrolment between 18 and 25 years; age at the first dose between 2 and 12 months of life or between 10.5 and 12.5 years. Time restrictions with regard to the age at the first dose arose from the fact that in Italy the vaccination against HBV became mandatory in 1991 for two cohorts of people (infants and adolescents). In this respect, the Italian law of 1991 was ahead of all other countries in the fight against HBV. The vaccination schedule was composed of 3 doses, given at 3, 5 and 11 months of life in infants and at time 0, 1 and 6 months later in adolescents^31^. In 2004, the vaccination of the catch-up cohort of adolescents was discontinued and maintained only in infants^41^.

### Laboratory testing

A commercial chemiluminescent microparticle immunoassay (CMIA) was used up the end of year 2017 to measure the titre of anti-hepatitis B surface (*s*) antigens (anti-HBs). Afterwards, the reference clinical microbiology laboratory changed the instrumentations and adopted a different commercial kit. The novel procedure uses a chemiluminescent immunoassay (CLIA) named LIAISON^®^ *anti-HBs plus* by Sorin (Saluggia, Italy). A negativity cut-off was set at 3 IU/L, without the possibility to know lower values. For this reason, values indicated as <3 IU/L were actually processed as 3 IU/L. Similarly, the method does not provide values higher than 1000 IU/L and, therefore, for a statistical evaluation of the antibody titre, they were processed as equal to 1000 IU/L. Antibodies titres higher than 10 IU/L were considered as protective, according to international standards^42^. However, it has to be considered that, as shown by current evidence, many of those with anti-HBs titre <10 IU/L respond optimally to the booster dose, supporting a strong immunological memory against HBV^43–45^, so that several authors question the aforementioned threshold and argue that a lowering of the threshold should be reconsidered^44,46,47^. In addition, all participants were tested for both HBcAb and HBsAg.

### Statistical analysis and legal aspects

Descriptive analysis was performed using the median, mean, geometric mean, standard deviation and geometric standard deviation for continuous variables, and absolute and relative frequencies for categorical variables. To allow the calculation of the geometric mean titre (GMT), a value of 0.5 IU/L was assigned arbitrarily in the case of undetectable concentrations. Differences in the distribution of categorical variables were assessed by means of Pearson’s χ^2^ test, while for continuous variables t tests were used; p-values and 95% confidence intervals were reported. We looked after the effect of the abovementioned variables on the long-term protection in each of the two considered cohorts (Cohort 1: infants; Cohort 2: adolescents), by means of a single-step logistic regression analysis. Age at first dose of vaccine, time between doses, follow-up duration and date of first dose administration and analysis were entered as independent variables. Adjusted odds ratios (AORs) and relative 95% confidence interval, B coefficients and *p* values were reported. Since our purpose was not to assess the effect of variables between groups, two different analyses were conducted. Statistical significance was set at *p* < 0.05. IBM SPSS version 28 was used for the statistical analyses. The research was based on data gathered during health surveillance, therefore evaluation by an ethics committee was not required. However, all subjects submitting to health surveillance signed a privacy document permitting the elaboration and publication of anonymous data. The collection of data was conducted following the principles of the Declaration of Helsinki, according to current national legislation and in compliance with the protection of personal data.

### Reporting summary

Further information on research design is available in the Nature Research Reporting Summary linked to this article.

## Supplementary information

REPORTING SUMMARY

## Data Availability

The raw data were generated at Padua University’s Occupational Health Surveillance Medical centre. Derived data supporting the findings of this study are available on request from the corresponding author exclusively for non-commercial use and under a Data Usage Agreement.
