# Supplementary material for: Factors influencing long-term persistence of anti-HBs after hepatitis B vaccination
Source: NPJ Vaccines. 2022 Dec 26;7:173. doi: 10.1038/s41541-022-00596-5 (PMC9792585; doi:10.1038/s41541-022-00596-5)
Supplement: Supplementary file 1 — REPORTING SUMMARY [file 41541_2022_596_MOESM1_ESM.pdf]

nature portfolio

Corresponding author(s): DBPR NPJVACCINES-02321R

Last updated by author(s): Oct 11, 2022

Reporting Summary

Nature Portfolio wishes to improve the reproducibility of the work that we publish. This form provides structure for consistency and transparency in reporting. For further information on Nature Portfolio policies, see our Editorial Policies and the Editorial Policy Checklist.

Statistics

For all statistical analyses, confirm that the following items are present in the figure legend, table legend, main text, or Methods section.

n/a	Confirmed	
☐	☒	The exact sample size (*n*) for each experimental group/condition, given as a discrete number and unit of measurement
☐	☒	A statement on whether measurements were taken from distinct samples or whether the same sample was measured repeatedly
☐	☒	The statistical test(s) used AND whether they are one- or two-sided<br>*Only common tests should be described solely by name; describe more complex techniques in the Methods section.*
☐	☒	A description of all covariates tested
☐	☒	A description of any assumptions or corrections, such as tests of normality and adjustment for multiple comparisons
☐	☒	A full description of the statistical parameters including central tendency (e.g. means) or other basic estimates (e.g. regression coefficient) AND variation (e.g. standard deviation) or associated estimates of uncertainty (e.g. confidence intervals)
☐	☒	For null hypothesis testing, the test statistic (e.g. *F*, *t*, *r*) with confidence intervals, effect sizes, degrees of freedom and *P* value noted<br>*Give P values as exact values whenever suitable.*
☒	☐	For Bayesian analysis, information on the choice of priors and Markov chain Monte Carlo settings
☒	☐	For hierarchical and complex designs, identification of the appropriate level for tests and full reporting of outcomes
☒	☐	Estimates of effect sizes (e.g. Cohen's *d*, Pearson's *r*), indicating how they were calculated

Our web collection on statistics for biologists contains articles on many of the points above.

Software and code

Policy information about availability of computer code

Data collection	MS Excel
Data analysis	IBM SPSS version 28

For manuscripts utilizing custom algorithms or software that are central to the research but not yet described in published literature, software must be made available to editors and reviewers. We strongly encourage code deposition in a community repository (e.g. GitHub). See the Nature Portfolio guidelines for submitting code & software for further information.

Data

Policy information about availability of data

All manuscripts must include a data availability statement. This statement should provide the following information, where applicable:

- Accession codes, unique identifiers, or web links for publicly available datasets
- A description of any restrictions on data availability
- For clinical datasets or third party data, please ensure that the statement adheres to our policy

Derived data supporting the findings of this study are available on request from the corresponding author.

Human research participants

Policy information about studies involving human research participants and Sex and Gender in Research.

Reporting on sex and gender	Data on age, sex, date of vaccine administration, as well as blood samples were collected.
Population characteristics	Students and resident doctors attending the Medical School of Padua University, Italy
Recruitment	The following inclusion criteria were considered: being born in Italy from HBsAg-negative mother, having received a three dose schedule vaccination against HBV as witnessed by a certificate issued by the Public Health Office, age at time of enrolment between 18 and 25 years; age at the first dose between 2 and 12 months of life or between 10.5 and 12.5 years.
Ethics oversight	The research was based on data gathered during health surveillance, therefore evaluation by an ethics committee was not required. However, all subjects submitting to health surveillance signed a privacy document permitting the elaboration and publication of anonymous data. The collection of data was conducted following the principles of the Declaration of Helsinki, according to current national legislation and in compliance with the protection of personal data.

Note that full information on the approval of the study protocol must also be provided in the manuscript.

Field-specific reporting

Please select the one below that is the best fit for your research. If you are not sure, read the appropriate sections before making your selection.

☒ Life sciences ☐ Behavioural & social sciences ☐ Ecological, evolutionary & environmental sciences

For a reference copy of the document with all sections, see nature.com/documents/nr-reporting-summary-flat.pdf

Life sciences study design

All studies must disclose on these points even when the disclosure is negative.

Sample size	Data were collected at the time of the first occupational health surveillance medical examination between January 2004 and December 2020. Routine detection of anti-HBs titre in serum before employment has been recommended for healthcare workers and students attending healthcare-related courses by the National Vaccine Prevention Plan (PNPV).
Data exclusions	The following inclusion criteria were considered: being born in Italy from HBsAg-negative mother, having received a three dose schedule vaccination against HBV as witnessed by a certificate issued by the Public Health Office, age at time of enrolment between 18 and 25 years; age at the first dose between 2 and 12 months of life or between 10.5 and 12.5 years. Time restrictions with regard to the age at the first dose arose from the fact that in Italy the vaccination against HBV became mandatory in 1991 for two cohorts of people (infants and adolescents).
Replication	Data on age, sex, date of vaccine administration, as well as blood samples were collected. A commercial chemiluminescent microparticle immunoassay (CMIA) was used up the end of year 2017 to measure the titre of anti-hepatitis B surface (s) antigens (anti-HBs). Afterwards, the reference clinical microbiology laboratory changed the instrumentations and adopted a different commercial kit. The novel procedure uses a chemiluminescent immunoassay (CLIA) named LIAISON® anti-HBs plus by Sorin (Saluggia, Italy).
Randomization	NA
Blinding	NA

Reporting for specific materials, systems and methods

We require information from authors about some types of materials, experimental systems and methods used in many studies. Here, indicate whether each material, system or method listed is relevant to your study. If you are not sure if a list item applies to your research, read the appropriate section before selecting a response.

Materials & experimental systems

n/a	Involved in the study
☒	☐ Antibodies
☒	☐ Eukaryotic cell lines
☒	☐ Palaeontology and archaeology
☒	☐ Animals and other organisms
☒	☐ Clinical data
☒	☐ Dual use research of concern

Methods

n/a	Involved in the study
☒	☐ ChIP-seq
☒	☐ Flow cytometry
☒	☐ MRI-based neuroimaging
